# End-of-life care at welfare evacuation centers following the 2024 Noto peninsula earthquake

**DOI:** 10.1017/S1478951525100308

**Published:** 2025-06-23

**Authors:** Momoka Yamamura, Ryo Ikeguchi, Makoto Kosaka, Toshiki Abe, Tianchen Zhao, Chika Yamamoto, Michioki Endo, Toyoaki Sawano, Kazuko Ishikawa, Nobuaki Moriyama, Akihiko Ozaki, Masaharu Tsubokura, Hiroyuki Beniya

**Affiliations:** 1 Department of Radiation Health Management, Fukushima Medical University School of Medicine, Fukushima, Japan; 2 Orange Home-Care Clinic, Fukui, Japan; 3 Department of Surgery, Hyogo Prefectural Awaji Medical Center, Hyogo, Japan; 4 Department of Surgery, Jyoban Hospital of Tokiwa Foundation, Fukushima, Japan; 5 Guruntobi, Fujisawa City, Kanagawa, Japan; 6 Department of Public Health, Fukushima Medical University School of Medicine, Fukushima, Japan; 7 Breast and Thyroid Center, Jyoban Hospital of Tokiwa Foundation, Fukushima, Japan

**Keywords:** End-of-life care, Palliative care, Welfare evacuation center, The 2024 Noto Peninsula earthquake, Disaster-related death

## Abstract

**Objective:**

To examine the challenges and practical realities of providing end-of-life care in welfare evacuation centers following the Noto Peninsula earthquake in Japan, and to identify lessons for improving disaster preparedness in similar settings.

**Case presentations:**

Case 1: A man in his late 90s was transferred to a welfare evacuation center after contracting COVID-19 in a general shelter. He arrived with fever and marked physical decline. Acetaminophen was administered to relieve his fever and provide comfort. His condition gradually worsened, and eight days after arriving at the evacuation shelter, he died peacefully while being closely observed by medical staff. Case 2: A man in his 60s with a history of smoking and alcohol use was found bedridden and incontinent at home and was subsequently moved to a welfare evacuation center. Two days after evacuation, he complained of leg and back pain, which was suspected to be due to arterial occlusion. He was monitored and provided with supportive care at the center, however, pain control remained inadequate. Four days after evacuation, he was found in respiratory arrest and was confirmed dead.

**Conclusion:**

These cases underscore the need for establishing unified guidelines and external support frameworks for end-of-life care in disaster settings. In a disaster-prone country like Japan, scenario-based training and the integration of trained volunteers are essential to ensuring dignified care for vulnerable evacuees.

## Introduction

Japan has entered a super-aged society, with increasing demands for medical care and long-term care for the elderly (Mathieu 2024). In this context, concepts such as living wills and Advance Care Planning (ACP) have gained prominence, emphasizing the importance of respecting individual preferences for end-of-life care. ACP is defined as a process of discussing and planning future medical care and treatment preferences with patients, their families, and healthcare teams, supporting patient-centered decision-making in anticipation of future changes in health status (Hannon et al. 2012).

End-of-life care aims to provide comfort and maintain quality of life while respecting the patient’s wishes and dignity. In addition to physical symptom management, psychological and social care are crucial, as terminal patients often experience various psychological states in accepting death. Inadequate communication may lead to unmet information needs, a lack of shared decision-making, and distrust in healthcare providers, potentially causing significant psychological distress (Boyle et al. 2005; Devi 2011; Tsuboi et al. 2022a).

Japan frequently experiences natural disasters that necessitate evacuation, which can adversely affect the health of vulnerable populations, including those with pre-existing conditions, disabilities, and the elderly (Chihoko Aoki 2011; Tsuboi et al. 2022a, 2022b; Yoshida et al. 2023; Yuna et al. 2023). To protect these vulnerable individuals, welfare evacuation centers were established following the 1995 Great Hanshin Earthquake (Hiroaki 2020).

Recently, there has been an increasing need for hospice and palliative care at welfare evacuation centers. This trend is driven by both proactive and reactive reasons. Proactively, providing care in familiar settings near evacuated family members offers psychological comfort and allows patients to spend their final moments peacefully in their hometowns. Reactively, welfare evacuation centers can provide basic care to alleviate the burden on overwhelmed medical facilities during disasters (Cabinet Office GoJ 2024; WHO 2018). However, their role in end-of-life care is not widely recognized, and most municipal guidelines do not address its implementation (Wang et al. 2016).

On January 1, 2024, a magnitude 7.6 earthquake struck the Noto region of Ishikawa Prefecture. The earthquake caused extensive damage to traditional wooden structures, with over 60,000 buildings damaged and 240 reported deaths as of February 5, 2024 (Wang et al. 2013). On January 8, a welfare evacuation center was established within a group home in Wajima City, accommodating approximately 30 evacuees until the end of March. That evacuation center was operated primarily by volunteer nurses and others gathered from across the country, and it continued for about three months with volunteers rotating on a weekly basis. We report two cases of end-of-life care experienced at this welfare evacuation center.

## Case presentations

Case 1: A male patient in his late 90s was evacuated to a general shelter after the earthquake, where he contracted COVID-19. On January 9, the couple was moved to a welfare evacuation center, where they were placed in a private room and cared for by nurses implementing infection control measures. The patient exhibited fever and significant physical debilitation upon arrival. At the welfare evacuation center, only antipyretic treatment with acetaminophen was administered, with no other medical interventions per the patient’s wishes. After discussions between the physician, patient, and family, it was agreed to respect the patient’s wishes and provide minimal antipyretics for comfort. On January 17, food intake nearly ceased completely. Early on January 18, at around 4 AM, the night nurse observed changes in the patient’s breathing, including jaw breathing and occasional apnea. The physician arrived promptly and remained at the patient’s bedside. Despite being unable to speak, the patient expressed gratitude and awareness of the nurses and physician through his labored breaths. By 5 AM, his breathing became progressively calmer with longer periods of apnea, eventually leading to respiratory arrest.

Case 2: A male patient in his 60s with a history of smoking and alcohol use experienced a decline in health around the end of 2023. On January 11, 2024, one nurse and two doctors from the center went to the patient’s home. They found the patient bedridden near the kitchen, incontinent of urine and feces. Unable to walk, staff carried the patient to the welfare evacuation center. Around January 13, the patient complained of lower back pain, which was treated with position changes and loxoprofen sodium administration, leading to gradual improvement. Initially thought to be postural, the leg and back pain, combined with the patient’s history and purple discoloration of the lower extremities, suggested arterial occlusion. When hospital transfer was proposed, the patient refused, preferring to remain at the evacuation center. Consideration was given to transferring the patient to medical facilities in neighboring Fukui or Toyama prefectures, but this course of action was temporarily abandoned due to poor road conditions. In this situation, with limited ability to consult external medical staff, decisions were left to the on-site physicians. It was decided to monitor the patient’s condition. In the evening of January 14, the patient again complained of foot and back pain, and pain medication was administered. At around 3 AM on January 15, the patient requested a bed position change due to back pain. When staff checked again 20 minutes later, the patient was found in respiratory arrest, and death was subsequently confirmed.

## Discussion

These cases illustrate the practice of end-of-life care in a welfare evacuation center setting, where both medical and human resources are less than ideal. One case demonstrates the potential to provide acceptable end-of-life care, while the other highlights various constraints inherent in hastily established systems.

Two main factors contributed to the successful end-of-life care in Case 1: First, the introduction of volunteers from outside the disaster area was crucial. When disaster victims who have experienced psychological trauma become rescue workers, they may face a double burden, leading to mental health issues and difficulties in providing sustained, high-quality care (Benedek et al. 2007; Peerboom et al. 2023; Rietze and Stajduhar 2015). In this case, by rapidly introducing volunteers, primarily healthcare professionals, to operate the evacuation center, it was possible to maintain the mental health of support staff while providing attentive care tailored to evacuees’ needs. In Case 1, staff were able to prepare the patient’s preferred meals and conduct regular nightly health checks, thus providing care that aligned closely with individual preferences. Second, thorough communication with evacuees contributed to the success. Communication in end-of-life care has been shown to play a crucial role in helping elderly individuals and their families organize their thoughts about future end-of-life care and prepare for decision-making (Izumi 2017; Kerr et al. 2020). Physicians and nurses play a role in conveying the importance of ACP and supporting decision-making through communication with patients and families (Cybulska and Rucinski 1989; Foy et al. 2010). In Case 1, discussions between the evacuee, family, and physician resulted in respecting the patient’s wish to follow a natural course with minimal medical intervention, supporting the patient’s desired end-of-life experience.

However, there are limitations to end-of-life care in welfare evacuation centers. Case 2 illustrates a situation where adequate medical care could not be provided due to limited medical resources in the surrounding area, a lack of easily accessible external support for on-site physicians to consult, and insufficient communication with the patient and family. In this case, with limited information about the patient’s medical history, on-site physicians were forced to make decisions with limited medical resources and information. Additionally, earthquake damage to roads connecting to neighboring prefectures increased travel times significantly, making patient transfer itself a risk (Wahlström 2015). The lack of accessible consultants further complicated the situation. Interactive communication among physicians involved in patient management is crucial for patients’ decision-making, and the introduction of external consultation services for on-site physicians during disasters is necessary (Khorram-Manesh et al. 2015; Plagg et al. 2023). While acute patients may face a binary choice between transfer or on-site end-of-life care, chronic patients present more complex decision-making challenges, and relying solely on physician judgment is not ideal for either patients or doctors. In Case 2, while the patient initially refused transfer, their and their family’s intentions regarding potential deterioration were unclear, leading to more passive life-saving measures compared to Case 1. Although human resources are limited during disasters, this case suggests that communication directly impacts patient health outcomes.

The importance of integrating palliative care and symptom relief into responses to humanitarian emergencies and crises has been highlighted by international organizations (Organization 2018). However, practical countermeasures for providing end-of-life care in welfare evacuation centers remain insufficient. Given that welfare evacuation centers housing care-dependent evacuees may encounter end-of-life situations, and considering Japan’s frequent exposure to disasters, it is necessary to establish unified end-of-life care guidelines and conduct regular scenario-based training.

Moreover, the deaths of these two evacuees may be considered disaster-related deaths, partly caused by changes in the living environment due to the earthquake. In the Great East Japan Earthquake and Fukushima Daiichi Nuclear Power Plant accident of March 11, 2011, 2,333 disaster-related deaths were reported, far exceeding the 1,614 direct disaster deaths (Yoshida et al. 2023). Previous literature suggests that secondary health impacts from evacuation may be equal to or greater than the impacts of the disaster itself (Kawashima et al. 2023; Kitazawa et al. 2023; Nomura et al. 2013; Sawano et al. 2023; Sonoda et al. 2019; Yamamura et al. 2024). This indicates the need to address not only the optimization of end-of-life care in welfare evacuation centers but also the minimization of secondary health impacts from evacuation to prevent disaster-related deaths.

## Conclusion

Report examines 2 end-of-life care cases at a welfare evacuation center following the 2024 Noto earthquake. Acceptable care is possible in non-ideal settings by integrating external resources and maintaining evacuees’ pre-disaster lifestyles. One case demonstrated success, while the other revealed system limitations. The experience highlights the need for external support systems, unified guidelines, and regular training for end-of-life care in disaster situations.
